# Activation of the Rat α1β2ε GABA_A_ Receptor by Orthosteric and Allosteric Agonists

**DOI:** 10.3390/biom12070868

**Published:** 2022-06-21

**Authors:** Allison L. Germann, Ariel B. Burbridge, Spencer R. Pierce, Gustav Akk

**Affiliations:** Department of Anesthesiology, Washington University School of Medicine, St. Louis, MO 63110, USA; germanna@wustl.edu (A.L.G.); arielburbridge@wustl.edu (A.B.B.); spencerp@wustl.edu (S.R.P.)

**Keywords:** GABA_A_ receptor, activation, potentiation, orthosteric agonist, allosteric agonist

## Abstract

GABA_A_ receptors are a major contributor to fast inhibitory neurotransmission in the brain. The receptors are activated upon binding the transmitter GABA or allosteric agonists including a number of GABAergic anesthetics and neurosteroids. Functional receptors can be formed by various combinations of the nineteen GABA_A_ subunits cloned to date. GABA_A_ receptors containing the ε subunit exhibit a significant degree of constitutive activity and have been suggested to be unresponsive to allosteric agents. In this study, we have characterized the functional properties of the rat α1β2ε GABA_A_ receptor. We confirm that the α1β2ε receptor exhibits a higher level of constitutive activity than typical of GABA_A_ receptors and show that it is inefficaciously activated by the transmitter and the allosteric agonists propofol, pentobarbital, and allopregnanolone. Manipulations intended to alter ε subunit expression and receptor stoichiometry were largely without effect on receptor properties including sensitivity to GABA and allosteric agonists. Surprisingly, amino acid substitutions at the conserved 9’ and 6’ positions in the second transmembrane (TM2) domain in the ε subunit did not elicit the expected functional effects of increased constitutive activity and resistance to the channel blocker picrotoxin, respectively. We tested the accessibility of TM2 residues mutated to cysteine using the cysteine-modifying reagent 4-(hydroxymercuri)benzoic acid and found a unique pattern of water-accessible residues in the ε subunit.

## 1. Introduction

The γ-aminobutyric acid type A (GABA_A_) receptor is an anion-conducting ion channel that plays a major role in mediating ionotropic inhibition in the central nervous system. The receptor is normally gated by the transmitter GABA, but it can also be directly activated or its activity potentiated by taurine, neurosteroids, and a number of sedatives and anesthetics [1]. The GABA_A_ receptor is a pentameric protein of homologous subunits, most frequently composed of two α subunits, two β subunits, and either a γ or a δ subunit; however, the α and β subunits can also assemble with the ε subunit to form a functional receptor with distinct properties [2,3]. The primary structure of the ε subunit most closely resembles that of mammalian γ subunits [2,3,4,5]. Expression of the ε subunit is restricted to a limited number of brain regions, including the amygdala, subthalamic nucleus, thalamus, hypothalamic nuclei, and the hilus of the hippocampus [2,3,5]. There is also evidence of ε subunit expression in peripheral tissue including the heart, pancreas, spleen, and prostate [3,4]. The physiological significance of the ε subunit and its role in normal brain function are demonstrated by the findings that nonsense and missense mutations in the ε subunit are linked to epileptic phenotypes [6].

Co-assembly of the ε subunit with α and β subunits produces receptors with a higher than typical level of constitutive activity [7,8,9,10]. The ε-containing GABA_A_ receptors also display slow deactivation and desensitization in response to pulses of GABA [11]. The pharmacological properties of ε subunit-containing receptors have not been fully established. Specifically, there has been some controversy regarding sensitivity to GABAergic anesthetics such as pentobarbital and propofol for which some studies [2,12] but not others [3] have reported the lack of a modulatory effect. A later study [13] proposed that differences in the level of ε subunit expression, resulting in receptor isoforms with different stoichiometries, may account for these conflicting findings.

The goal of the present study was to quantitatively characterize the activation and potentiation parameters of the α1β2ε GABA_A_ receptor. We have found that the α1β2ε receptor exhibits unusually high constitutive activity and is only weakly activated by the transmitter GABA and the allosteric agonists propofol, pentobarbital, and the neurosteroid allopregnanolone (3α5αP). Changes in the relative amount of ε subunit cRNA injected had minimal effect on receptor properties. We also present evidence that the ε subunit is unique in how mutations to conserved hydrophobic and polar residues in the second transmembrane (TM2) domain affect activation by GABA and inhibition by picrotoxin.

## 2. Materials and Methods

### 2.1. Receptors and Expression

The receptors were expressed in oocytes from *Xenopus laevis* (African clawed frog). The oocytes were purchased from Xenopus 1 (Dexter, MI) as quarter ovaries. The ovaries were digested in a 2% *w*/*v* (mg/mL) solution of collagenase A in ND96 solution (96 mM NaCl, 2 mM KCl, 1.8 mM CaCl_2_, 1 mM MgCl_2_, 5 mM HEPES; pH 7.4) with 100 U/mL penicillin and 100 μg/mL streptomycin while shaking at 150 RPM for 30 to 40 min at 37 °C. The oocytes were then rinsed in ND96 solution and incubated in ND96 solution with supplements (2.5 mM Na pyruvate, 100 U/mL penicillin, 100 μg/mL streptomycin, 50 μg/mL gentamycin) at 15 °C for at least 4 h prior to injection of cRNA.

The cDNAs for rat α1 (accession no. NM_183326.2), β2 (NM_012957.2), and ε subunits (NM_023091.1) were subcloned into the pcDNA3 vector and linearized using either XbaI or ApaI (NEB labs, Ipswich, MA, USA). The cDNA for the ε subunit was provided by Dr. M. Garret (University of Bordeaux). The ε subunit clone used in this study did not contain the ~400 Pro/Glx repeat in the extracellular domain [4,5,14]. The cDNAs for mutant subunits were synthesized and cloned into pcDNA3 by Twist BioScience (San Francisco, CA, USA). The α1(V257C) and α1(T261C) subunits were made on the basis of the human α1 sequence (NM_000806.5) that differs from the rat sequence by one extra amino acid residue in the extracellular domain.

The cRNAs were generated using mMessage mMachine T7 and Poly(A) Tailing Kit (Invitrogen, Waltham, MA, USA). The oocytes were injected with a total of 3 to 12 ng cRNA per oocyte. The cRNA ratio was 1:1:5 (α:β:ε) unless specified otherwise. Some experiments were conducted on oocytes injected with 1:1:1 or 1:1:10 (α:β:ε) cRNA ratios. Additional control experiments were done on α1β2 receptors (cRNA ratio 1:1), and on wild-type and mutant α1β2γ2L receptors (1:1:5). The injected oocytes were stored in ND96 with supplements at 15 °C for 2–3 days prior to conducting electrophysiological recordings.

### 2.2. Electrophysiology

The electrophysiological recordings were conducted at room temperature using standard two-electrode voltage clamp. The borosilicate pipets (G120F-4, Warner Instruments, Hamden, CT, USA) were filled with 3 M KCl. The oocytes were placed in the recording chamber (RC-1Z, Warner Instruments) where they were voltage-clamped at −60 mV. Bath and drug solutions were gravity applied to the recording chamber at a rate of 5–8 mL/min from 30 mL syringes with glass luer slips via Teflon tubing. Solutions were switched manually using a medium pressure 6-port bulkhead valve (IDEX Health and Science, Rohnert Park, CA, USA).

In a typical experiment, the bath solution was applied for 10–20 s to establish baseline activity, followed by a 10–60 s application of a drug solution to assess the peak response, or a 5 min drug application to assess the steady-state response. After each application, bath solution was applied for 2–5 min to verify recovery to baseline. Since the current responses under several conditions were small (<20 nA), special care was taken to avoid contamination and false-positive responses. For example, test applications of bath or low GABA were done to verify the lack of current response or constant current response from all syringes. This was done as appropriate on each day of recordings.

Current responses were amplified with OC-725C (Warner Instruments, Hamden, CT, USA) or Axoclamp 900A (Molecular Devices, Sunnyvale, CA, USA) amplifiers, digitized with a Digidata 1200 or 1320 series digitizer (Molecular Devices), and stored electronically using pClamp (Molecular Devices).

### 2.3. Data Analysis

The current traces were analyzed using Clampfit (Molecular Devices) to determine the peak and steady-state amplitudes. If a current response failed to reach steady-state (defined as ΔI < 2% during the last 20 s of agonist application), the steady-state current level was estimated by the exponential fitting of the current response.

Descriptive analysis of receptor activation by agonist was done by normalizing the response amplitudes to the peak response to saturating agonist or the combination of GABA + propofol in the same cell, and fitting the normalized responses to Equation (1):

(1) $${Y=Y}_{\min}{+(Y}_{\max}{–Y}_{\min})\frac{{\left[\mathrm{agonist}\right]}^{n_{H}}}{{\left[\mathrm{agonist}\right]}^{n_{H}}{+\mathrm{EC}}_{50}{}^{n_{H}}}$$

where Y_min_ and Y_max_ are the low- and high-concentration asymptotes, [agonist] is the concentration of agonist used, EC_50_ is the concentration of agonist producing a half-maximal effect, and n_H_ is the Hill slope. The fitting parameters were determined for each cell individually and are presented as mean ± S.D.

Mechanistic analysis of receptor activity was done in the framework of a concerted transition model [15,16]. The raw peak amplitudes were converted to values of probability of being in the active state (P_A_) by comparing the peak responses to the agonist to the current response produced by 200 μM picrotoxin that is expected to block all surface GABA_A_ receptors (P_A_ approaches 0), and the peak response to 1 mM GABA + 10 μM propofol that is expected to activate all surface GABA_A_ receptors (P_A_ approaches 1). The approach has been described in detail previously [17].

The P_A_ data were fitted to the state equation [15,16]:

(2) $$P_{A}=\frac{1}{1+L{\left[\frac{{1+[\mathrm{agonist}]/K}_{R,\mathrm{agonist}}}{{1+[\mathrm{agonist}]/(K}_{R,\mathrm{agonist}}c_{\mathrm{agonist}})}\right]}^{N_{\mathrm{agonist}}}}$$

where [agonist] is the concentration of agonist, K_R,agonist_ is the equilibrium dissociation constant for the agonist in the resting receptor, *c*_agonist_ is the ratio of the equilibrium dissociation constant for the agonist in the active receptor to K_R_, and N_agonist_ is the number of agonist binding sites. L is set by the level of background activity and is calculated as L = (1 − P_A,background_)/P_A,background_. The fitting parameters were determined for each cell individually, and are presented as mean ± S.D.

Statistical analyses (*t*-test or ANOVA with Dunnett’s or Bonferroni’s correction, as appropriate) were done using Excel 2016 (Microsoft, Redmond, WA, USA) or Stata 12.1 (StataCorp, College Station, TX, USA). Curve fitting was done using Origin 2020 (OriginLab Corp., Northampton, MA, USA).

### 2.4. Cysteine Modification Experiments

To identify the residues lining the ion permeation pathway, we employed the scanning cysteine accessibility method [18,19]. In this approach, residues in the region of interest are individually mutated to cysteine, and the mutant receptors are exposed to a small, charged sulfhydryl-specific reagent. Covalent labeling of a cysteine residue, and by extension its aqueous accessibility, are deduced from irreversible alteration of receptor function. Cysteine modification was tested on wild-type (control) and cysteine-mutant α1β2γ2L and α1β2ε receptors by measuring and comparing the peak amplitudes of responses to 10–20 s applications of saturating GABA before and after exposure to a 1 min application of 100 μM of the cysteine-modifying reagent 4-(hydroxymercuri)benzoic acid sodium salt (pCMB) or pCMB + saturating (10 µM–1 mM) GABA. Prior to exposure to pCMB, up to five applications of saturating GABA were done until a stable (<10% change) response was obtained. All drug applications were followed by at least 4 min washouts in ND96. Modification of the introduced cysteines was determined by comparing the immediate post- and pre-pCMB exposure peak amplitudes of responses to GABA. If a significant difference was observed, then the residue mutated to cysteine was considered to be accessible and labeled by pCMB.

### 2.5. Materials

The salts and HEPES used in the ND96 solution, GABA, pentobarbital, picrotoxin, and pCMB were purchased from Sigma-Aldrich (St. Louis, MO, USA). Propofol was purchased from MP Biomedicals (Solon, OH, USA). The steroid 3α5αP was purchased from Tocris Bioscience (Bristol, UK).

GABA was dissolved in ND96 to a concentration of 500 mM and stored in aliquots at −20 °C. Picrotoxin was dissolved in ND96 to a concentration of 2 mM and stored at 4 °C. The steroid 3α5αP was dissolved in DMSO at 10–20 mM and stored at room temperature. The stock solution of pentobarbital was made in ND96 to a concentration of 3 mM, pH-adjusted, and stored at room temperature. The stock solution of propofol was made in DMSO at 200 mM and stored at room temperature. The stock solution of pCMB was made in DMSO at 20 mM and stored at room temperature. Dilutions were made daily as needed to a maximal final concentration of DMSO of 0.5%, which has been shown to have no effect on holding current or GABA_A_ receptor responses to the transmitter [20].

## 3. Results

### 3.1. GABA Is a Weak Agonist of the α1β2ε GABA_A_ Receptor

**Figure 1:**
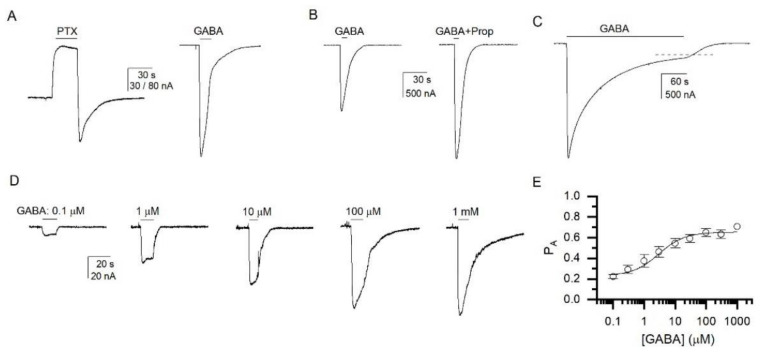
**Basic characterization of properties of the α1β2ε GABA_A_ receptor.** (**A**) Sample current responses to 200 µM picrotoxin (PTX) and 1 mM GABA. (**B**) Sample current responses to 1 mM GABA and 1 mM GABA + 10 µM propofol (Prop). In panels (**A**,**B**), the paired current responses are from the same cells. (**C**) Sample current response to a prolonged application of 1 mM GABA. The dashed line shows the fitted level of steady-state current. (**D**) Sample current responses to 0.1 µM, 1 µM, 10 µM, 100 µM, and 1 mM GABA. All traces are from the same cell. (**E**) The GABA concentration-response relationship. The data are expressed in the units of P_A_. The data points give means ± S.D. from seven cells. The curve is calculated using the mean K_R,GABA_ (4.2 µM) and *c*_GABA_ (0.399) values with the number of GABA binding sites constrained to 2.

We commenced the studies by conducting a basic characterization of activation of the α1β2ε receptor by the transmitter. The receptors were expressed in the cRNA injection ratio of 1:1:5 (α1:β2:ε). To gain insight into the extent of constitutive activity, we compared responses to GABA and the channel blocker picrotoxin. In 14 cells, the ratio of the response to 200 µM picrotoxin was −0.36 ± 0.20 (mean ± S.D.) of the response to 1 mM GABA. The ratio is negative to indicate the opposite direction of picrotoxin- and GABA-elicited currents. Sample current traces in the presence of picrotoxin and GABA are given in Figure 1A. The concentrations of picrotoxin and GABA used here are saturating based on previous reports [7,9]. The observed picrotoxin to GABA response ratio is similar to those reported previously [9,11,13].

Next, we compared the peak responses to saturating GABA applied alone or in the presence of 10 µM propofol (Figure 1B) or 1 µM 3α5αP. The relative peak responses to 1 mM GABA were 0.64 ± 0.11 (*n* = 8) of the response to 1 mM GABA + 10 µM propofol, and 0.67 ± 0.07 (*n* = 5) of the response to 1 mM GABA + 1 µM 3α5αP (*p* > 0.05 for the ratios; *t*-test). From the GABA to GABA + propofol, and picrotoxin to GABA response ratios, we calculate that the probability of being constitutively active (P_A,constitutive_) is 0.18 ± 0.08 (*n* = 14), and the peak probability of being in the active state (P_A_) in the presence of 1 mM GABA is 0.71 ± 0.09 (*n* = 8). In these calculations, we have assumed that exposure to picrotoxin blocks all constitutively-active surface receptors (P_A_ = 0), and that exposure to GABA + propofol activates all surface receptors (P_A_ = 1) [17].

Desensitization properties of the α1β2ε receptor were examined by exposing the cells to 5 min applications of 1 mM GABA. In five cells, the steady-state to peak ratio was 0.23 ± 0.17. Desensitization decay was fitted to sums of two exponentials. The fitted time constants were 16.1 ± 2.4 s (23%) and 155 ± 81 s (τ_weighted_ = 120 ± 54 s). A sample current trace is given in Figure 1C. The steady-state to peak ratio is similar to that in the α1β2γ2L receptor [21].

The GABA concentration-response relationship was measured by exposing cells expressing α1β2ε receptors to 0.1 µM to 1 mM GABA (Figure 1D). Fitting the concentration-response data for each cell individually with Equation (1) yielded an EC_50_ of 2.9 ± 1.7 µM and a Hill coefficient of 0.66 ± 0.12 (*n* = 7). The estimated EC_50_ is within the range of previously reported GABA EC_50_s (1–11 µM) for ε-containing receptors [2,3,7,9].

We also analyzed the GABA concentration-response data in the framework of a concerted transition model [15,16]. The response amplitudes were converted to P_A_ units as described previously [17]. Fitting the P_A_ data with Equation (2), with the number of GABA binding sites constrained to two [1], yielded a K_R,GABA_ of 4.2 ± 2.0 µM and a *c*_GABA_ of 0.399 ± 0.022. The summary of the concentration-response analysis is given in Figure 1E. The calculated ∆G provided by GABA to stabilize the active state is −1.09 ± 0.07 kcal/mol. For comparison, GABA provides −5.8 to −6.7 kcal/mol of activation energy in the α1β2γ2L receptor [22,23].

Receptors comprising α and β subunits are functional [24]. To verify the expression and incorporation of the ε subunit in surface receptors, we compared the properties of receptors expressed upon the injection of α1 and β2 subunits to the properties of receptors expressed upon the injection of α1, β2, and ε subunits. We have assumed that differences in the properties of α1β2 and nominal α1β2ε receptors indicate the presence of the ε subunit in the receptor complex.

**Figure 2:**
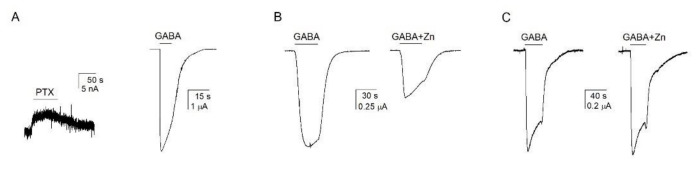
**Comparison of properties of α1β2 and α1β2ε GABA_A_ receptors.** (**A**) Sample current responses to 200 µM picrotoxin (PTX) and 1 mM GABA in a cell expressing α1β2 receptors. Analogous comparison for the α1β2ε receptor is given in Figure 1A. (**B**) Sample current responses to 3 µM GABA and 3 µM GABA + 3 µM ZnCl_2_ in a cell expressing α1β2 receptors. (**C**) Sample current responses to 3 µM GABA and 3 µM GABA + 3 µM ZnCl_2_ in a cell expressing α1β2ε receptors. In all panels, the paired recordings are from the same cells.

We conducted two tests. First, we compared the levels of constitutive activity and peak responses to saturating GABA. The P_A,constitutive_ and P_A,1mM GABA_ were estimated by comparing the amplitudes of current responses to 200 µM picrotoxin, 1 mM GABA, and 1 mM GABA + 10 µM propofol (see above; [17]). In the α1β2 GABA_A_ receptor, the estimated P_A,constitutive_ was 0.0013 ± 0.0019 (*n* = 11), and the P_A,1mM GABA_ was 0.59 ± 0.18 (*n* = 6). The P_A,constitutive_ in α1β2 and α1β2ε receptors are significantly different (*p* < 0.001; *t*-test). Sample traces showing responses to picrotoxin and GABA in the α1β2 receptor are given in Figure 2A.

In the second test, we measured the inhibitory effect of Zn^2+^. Ternary GABA_A_ receptors and specifically the αβε receptor are less sensitive to Zn^2+^ than the binary αβ receptor (IC_50_s at tens to hundreds of micromolar vs. sub-micromolar concentrations; [3,7,25]). Here, we compared the effect of 3 µM Zn^2+^ on currents elicited by an ~EC_50_ concentration of GABA in α1β2 and α1β2ε receptors. The peak and residual (at the end of a 30 s application) responses to GABA + Zn^2+^ were compared to control GABA responses measured before exposure to Zn^2+^. In the α1β2 receptor, exposure to Zn^2+^ reduced the peak response to 3 µM GABA (EC_54±14_) to 42 ± 20% of control and the residual response to 28 ± 19% (*n* = 5) of control. In contrast, in the nominal α1β2ε receptor the peak and residual responses to 3 µM GABA (EC_67±23_) in the presence of Zn^2+^ were 96 ± 9% and 79 ± 15% (*n* = 6) of control, respectively. The effects of Zn^2+^ on both peak and residual currents are significantly (*p* < 0.001) different in the α1β2 and α1β2ε receptors. Sample current responses to GABA and GABA + Zn^2+^ are shown in Figure 2B,C. We infer that the ε subunit is incorporated into receptor complexes and that this results in receptors with distinct properties.

### 3.2. Activation of the α1β2ε GABA_A_ Receptor by Allosteric Agonists

Some prior work has indicated that ε subunit-containing receptors may be insensitive to the potentiating effects of allosteric agents including neurosteroids and GABAergic anesthetics ([2] but see [3,7]). Here, we have investigated activation and potentiation of the α1β2ε receptor by the allosteric agonists propofol, 3α5αP, and pentobarbital.

**Figure 3:**
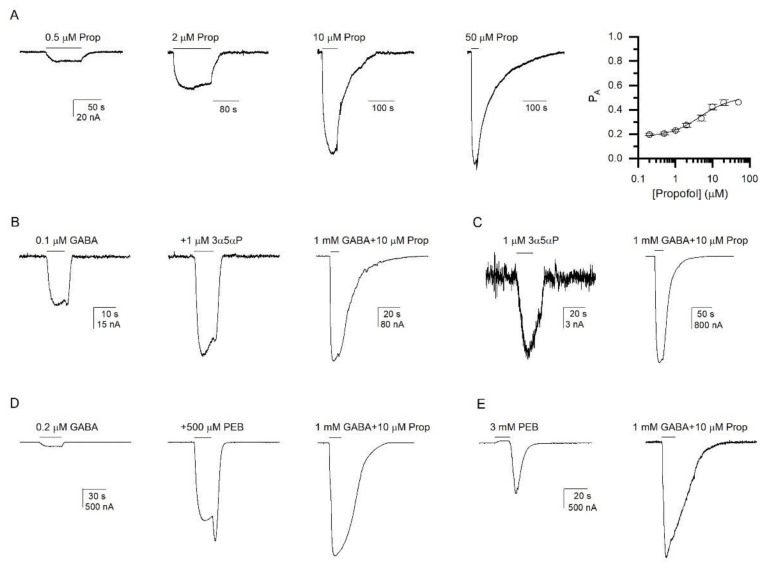
**Activation and potentiation of the α1β2ε GABA_A_ receptor by allosteric agonists.** (**A**) Sample current responses to 0.5, 2, 10, and 50 µM propofol (Prop). All traces are from the same cell. The right panel gives the propofol concentration-response relationship. The data are expressed in the units of P_A_. The data points give means ± S.D. from five cells. The curve is calculated using the mean K_R,propofol_ (4.1 µM) and *c*_propofol_ (0.771) values with the number of propofol binding sites constrained to 6. (**B**) Sample current responses to 0.1 µM GABA and 0.1 µM GABA + 1 µM 3α5αP. For comparison, a response to 1 mM GABA + 10 µM propofol (Prop) is given. All traces are from the same cell. (**C**) Sample current responses to 1 µM 3α5αP and 1 mM GABA + 10 µM propofol (Prop), from the same cell. (**D**) Sample current responses to 0.2 µM GABA and 0.2 µM GABA + 500 µM pentobarbital (PEB). For comparison, a response to 1 mM GABA + 10 µM propofol (Prop) is given. All traces are from the same cell. The amplitude of rebound current upon washout of GABA + PEB was used for analysis. (**E**) Sample current responses to 3 mM pentobarbital (PEB) and 1 mM GABA + 10 µM propofol (Prop), from the same cell. The amplitude of rebound current upon washout of PEB was used for analysis.

Cells expressing α1β2ε receptors were exposed to 0.2–50 µM propofol (Figure 3A). Fitting of the concentration-response data with Equation (1) yielded an EC_50_ of 4.4 ± 0.9 µM and a Hill coefficient of 1.22 ± 0.18 (*n* = 5). Mechanistic analysis (Equation (2)) of the propofol concentration-response data, with the number of propofol binding sites constrained to six [26], yielded a K_R,propofol_ of 4.1 ± 0.9 µM and a *c*_propofol_ of 0.771 ± 0.011. The calculated activation energy provided by propofol is −0.92 ± 0.05 kcal/mol. (We note that the calculated ∆G is not sensitive to the number of imposed binding sites for the agonist.) For comparison, propofol provides −5.3 to −6.5 kcal/mol of free energy change in the α1β2γ2L receptor [26,27]. Thus, the α1β2ε receptor is weakly activated by propofol.

Next, we investigated potentiation of GABA-elicited currents by the neurosteroid 3α5αP. Co-application of 1 µM 3α5αP enhanced the response to 0.1 µM GABA (EC_6_; P_A_ = 0.22) to 222 ± 13% (*n* = 5) of control (Figure 3B). In previous work, we have shown that high P_A,constitutive_ is associated with reduced potentiation but increased direct activation [28]. We, therefore, measured 3α5αP-induced direct activation of the α1β2ε receptor. The cells were exposed to 0.03 to 3 µM 3α5αP. For normalization purposes, each cell was additionally exposed to 1 mM GABA + 10 µM propofol. No meaningful current responses were observed in the presence of 0.03 to 0.3 µM 3α5αP (not shown). Consistent and robust currents were recorded only upon the application of 1 or 3 µM steroid, while the relative amplitudes were indistinguishable indicating that direct activation is saturated at 1 µM 3α5αP. The relative response to 1 µM 3α5αP was 2.0 ± 2.1% (*n* = 11) of the peak response to 1 mM GABA + 10 µM propofol, translating into a P_A,1 µM 3α5αP_ of 0.20 ± 0.02 (Figure 3C). From here, we could estimate the value of *c*_3α5αP_, assuming that the response to 1 µM steroid is saturating:

(3) $$c_{\mathrm{agonist}}={\left[\frac{{(1/P}_{A})-1}{L}\right]}^{{1/N}_{\mathrm{agonist}}}$$

where L reflects the level of constitutive activity (L = (1 − P_A,constitutive_)/P_A,constitutive_) and N_agonist_ is the number of steroid-binding sites. With the number of steroid-binding sites constrained to two [29,30], the calculated *c*_3α5αP_ is 0.951 ± 0.047, and the free energy change contributed by the steroid −0.06 ± 0.06 kcal/mol. This is drastically less than the free energy change in the α1β2γ2L GABA_A_ receptor (~−2.00 kcal/mol [31,32]). The 3α5αP direct activation data are in general agreement with potentiation data; the *c*_3α5αP_ of 0.951 predicts potentiation to 150% of control at 0.1 µM GABA. In sum, we infer that the neurosteroid 3α5αP is a very inefficacious agonist of the α1β2ε receptor.

Coapplication of 500 µM pentobarbital with 0.2 µM GABA (3 ± 1% of the response to 1 mM GABA + 10 µM propofol; P_A_ = 0.20 ± 0.01; *n* = 5) enhanced the response amplitude by 28 ± 5-fold (P_A_ = 0.85 ± 0.07). Sample current traces are shown in Figure 3D. Assuming that the potentiating effect of pentobarbital is saturated at 500 µM [7], the calculated (Equation (3)) *c*_pentobarbital_, with N_pentobarbital_ constrained to two [33], is 0.210 ± 0.057. The ∆G provided by pentobarbital to stabilize the active state is −1.88 ± 0.33 kcal/mol. Pentobarbital is therefore a weaker agonist of the α1β2ε than the α1β2γ2L receptor (∆G = −6.0 to −6.5 kcal/mol [31,33]). A complementary attempt to estimate the gating efficacy of pentobarbital was made from the analysis of α1β2ε receptors directly activated by pentobarbital. From the comparison of the rebound response upon the washout of 3 mM pentobarbital and the peak response to 1 mM GABA + 10 µM propofol (Figure 3E), we estimated that P_A,3 mM pentobarbital_ is 0.60 ± 0.11 (*n* = 6). Using Equation (3), we then calculated a *c*_pentobarbital_ of 0.384 ± 0.086, and ∆G_pentobarbital_ of −1.15 ± 0.27 kcal/mol. The value of *c*_pentobarbital_ calculated from direct activation data is greater than that calculated from potentiation data, implying lower efficacy. Potential explanations for this are that direct activation is not saturated at 3 mM pentobarbital and/or that the peak amplitude measured as the rebound current upon the termination of application of pentobarbital underestimates the true current response due to concurrent channel block.

### 3.3. Changes in the Expression of ε Subunit Have Minor Effect on Receptor Function

It has been proposed previously that the properties of the αβε receptor depend on the relative expression level of the ε subunit. Overexpression of the ε subunit has been associated with enhanced constitutive activity, increased sensitivity to GABA, and reduced sensitivity to the potentiating actions of anesthetics and neurosteroids [8,13].

Here, we compared the properties of α1β2ε receptors expressed under the injection ratios of 1:1:1, 1:1:5, and 1:1:10. We measured the levels of constitutive activity, GABA EC_50_s, peak P_A_ of saturating GABA, and P_A_ of the responses to 50 µM propofol or 1 µM 3α5αP. We estimate that the P_A,constitutive_ are 0.16 ± 0.12 (*n* = 10) and 0.31 ± 0.13 (*n* = 13) in cells injected with 1:1:1 and 1:1:10 cRNA (α:β:ε) ratios, respectively. As shown above, the P_A,constitutive_ is 0.18 ± 0.08 at 1:1:5 injection ratio. The P_A,constitutive_ at 1:1:10 injection ratio significantly (*p* < 0.05) differs from the P_A,constitutive_ at 1:1:5 and 1:1:1 injection ratios.

The GABA EC_50_s were 2.6 ± 1.2 µM (*n* = 5) at 1:1:1, 2.9 ± 1.2 µM at 1:1:5 (see above), and 2.4 ± 1.0 µM (*n* = 5) at 1:1:10 injection ratio. The estimated peak P_A,1 mM GABA_ were 0.63 ± 0.13 (*n* = 8), 0.71 ± 0.09 (see above), and 0.79 ± 0.05 µM (*n* = 6) at 1:1:1, 1:1:5, and 1:1:10 injection ratios, respectively. The differences in GABA EC_50_s and P_A,1 mM GABA_ are not statistically significant (*p* > 0.05).

The peak P_A_ in the presence of 50 µM propofol was 0.54 ± 0.18 (*n* = 6), 0.46 ± 0.09 (*n* = 5), and 0.66 ± 0.03 (*n* = 5) in cells injected at 1:1:1, 1:1:5, and 1:1:10 cRNA ratios, respectively. Note that the values include constitutive activity, so the higher constitutive P_A_ contributed to the higher P_A,50 µM propofol_ at 1:1:10.

The ratios of responses to 1 µM 3α5αP and 1 mM GABA + 10 µM propofol were indistinguishable for all injection ratios (1.3 ± 0.7%, 2.0 ± 2.1%, 1.5 ± 1.3% at 1:1:1, 1:1:5, and 1:1:10, respectively). The peak P_A_ in the presence of 1 µM 3α5αP was 0.17 ± 0.01 (*n* = 5) at 1:1:1, 0.20 ± 0.02 (see above) at 1:1:5, and 0.32 ± 0.01 (*n* = 5) at 1:1:10 injection ratio. The P_A,1µM 3α5αP_ were different (*p* < 0.01) for any pairwise comparison. The apparent increase in gating efficacy for 3α5αP is, however, almost fully due to the increase in P_A,constitutive_.

We also compared the potentiating effect of 3α5αP at different injection ratios. Coapplication of 1 µM 3α5αP with 0.1–0.3 µM GABA enhanced the current response to 313 ± 87% (*n* = 6), 222 ± 13% (*n* = 5), or 173 ± 19% (*n* = 5) of control at 1:1:1, 1:1:5, and 1:1:10 injection ratios, respectively. The effects observed at 1:1:1 and 1:1:10 injection ratios were significantly (*p* < 0.01) different.

The overall finding is that overexpression of the ε subunit leads to an increase in P_A,constitutive_ without affecting other tested activation parameters, including the efficacy of the transmitter GABA or the allosteric agonists propofol and 3α5αP. An increase in P_A,constitutive_ upon overexpression of the ε subunit has been reported previously [13]. This, however, was accompanied by a loss of apparent potentiation of low GABA responses by allosteric agonists including a neurosteroid. One potential explanation for this apparent discrepancy is that overexpression of the ε subunit in our hands leads to the presence of a class of surface receptors that are constitutively active and can be blocked by picrotoxin, but which do not respond to GABA or allosteric agonists.

### 3.4. Atypical Properties of the α1β2ε Receptor

**Figure 4:**
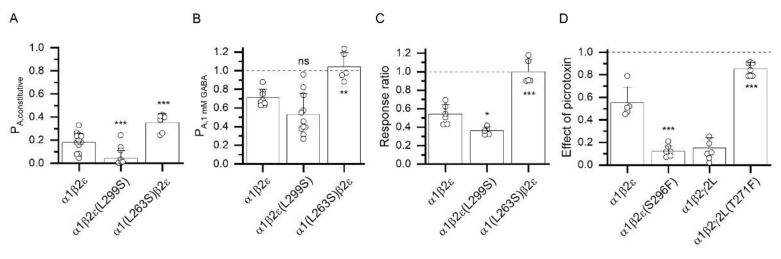
**Divergent consequences of homologous mutations in the TM2 regions of ε subunits and α1 or γ2L subunits.** (**A**) P_A,constitutive_ in α1β2ε, α1β2ε(L299S), and α1(L263S)β2ε receptors. (**B**) P_A,1 mM GABA_ in α1β2ε, α1β2ε(L299S), and α1(L263S)β2ε receptors. (**C**) Response ratio gives the ratio of peak responses to applications of 3 µM and 1 mM GABA in α1β2ε, α1β2ε(L299S), and α1(L263S)β2ε receptors. Lower values of the ratio are indicative of a right-shifted concentration-response relationship. (**D**) Effect of picrotoxin describes the ratio of responses to 1 mM GABA + 100 µM picrotoxin and 1 mM GABA tested before exposure to picrotoxin in α1β2ε and α1β2ε(S296F) receptors, and in α1β2γ2L and α1β2γ2L(T271F) receptors. Lower values of the ratio are indicative of higher sensitivity to channel blocker picrotoxin (1 indicates no effect). Statistical analysis was done using ANOVA with Dunnett’s post hoc correction (**A**–**C**) or *t*-test (**D**) comparing the properties of mutant receptors to the respective wild-type receptor. The ε(L299) and α1(L263) residues are homologous 9’ residues in TM2. The ε(S296) and γ2L(T271) residues are homologous 6’ residues in TM2. *, *p* < 0.05; **, *p* < 0.01; ***, *p* < 0.001; ns, not significant.

To gain further insight into the properties of the α1β2ε receptor, we examined the effects of mutations in the TM2 region of the ε subunit. First, we mutated the conserved 9’ leucine residue to serine (L299S in the ε subunit). In previous work on γ and δ subunit-containing receptors, the TM2-L9’S mutation has been shown to increase the level of constitutive activity and sensitivity to agonists while the magnitude of the effect is dependent on the number of mutated subunits [34,35,36]. In contrast, the α1β2ε(L299S) receptor did not demonstrate elevated constitutive activity or higher sensitivity to GABA. In the mutant receptor, the estimated P_A,constitutive_ was 0.06 ± 0.08 (*n* = 11), and the P_A,1 mM GABA_ was 0.53 ± 0.22 (*n* = 11), indicating significant decreases in constitutive activity (*p* < 0.001) and maximal P_A_ for GABA (*p* < 0.05). To gain initial insight into the position of the transmitter concentration-response relationship, we tested the ratio of peak responses to 3 µM and 1 mM GABA. In the α1β2ε receptor, this ratio is 0.54 ± 0.10 (*n* = 7). In the α1β2ε(L299S) receptor, the 3 µM/1 mM GABA response ratio was 0.36 ± 0.04 (*n* = 5), indicating a small right-shift, rather than the expected left-shift, in the GABA concentration-response relationship. The data are summarized in Figure 4A–C.

To verify the presence of the ε(L299S) subunit in surface receptors, we compared the P_A,constitutive_ and sensitivity to Zn^2+^ in α1β2 and α1β2ε(L299S) receptors. We reasoned that differences in the properties of α1β2 and nominal α1β2ε(L299S) receptors would serve as proof of the presence of the ε(L299S) subunit. The estimated P_A,constitutive_ in α1β2 and α1β2ε(L299S) (0.0013 and 0.06, respectively) were significantly (*p* < 0.05) different. In the α1β2ε(L299S) receptor, co-application of 3 µM Zn^2+^ with 3 µM GABA (EC_49±14_) reduced the peak and residual currents at the end of a 30 s drug application to 70 ± 12% and 41 ± 5% (*n* = 5) of control, respectively. The observed inhibitory effect of Zn^2+^ on the peak response in α1β2ε(L299S) is significantly (*p* < 0.05) different from its effect on the peak response in the α1β2 receptor (42); above). The effects on residual current were indistinguishable in α1β2ε(L299S) and α1β2 receptors.

We also measured the effect of the homologous α1(L263S) mutation on receptor properties (Figure 4A–C). The P_A,constitutive_ was increased to 0.35 ± 0.08 (*n* = 6) in the α1(L263S)β2ε receptor. This is significantly (*p* < 0.001) different from the level of constitutive activity in wild-type α1β2ε. The estimated P_A,1 mM GABA_ was 1.04 ± 0.15 (*n* = 5), and the 3 µM/1 mM GABA response ratio was 1.00 ± 0.13 (*n* = 5). The observed changes in receptor parameters are indicative of an increase in gating efficacy and are in agreement with previous data on how the TM2-L9’S mutation modifies GABA_A_ receptor function [34,36].

Second, we tested the effect of the ε(S296F) mutation on inhibition by picrotoxin. Substitution of this residue (TM2-6’) in other subunits has been shown to drastically reduce inhibition by picrotoxin; specifically, mutation of even a single 6’ residue to phenylalanine in the α1β2γ2 receptor has been shown to render the receptor resistant to picrotoxin [37,38].

The experiments were done by exposing a cell to short applications of 1 mM GABA separated by 4–5 min washes until a stable response to GABA was obtained. We then applied 100 µM picrotoxin for 10 s, followed by co-application of 1 mM GABA + 100 µM picrotoxin. The peak amplitude of the response to GABA + picrotoxin was compared to that of the control response to GABA alone applied before exposure to picrotoxin to calculate a response ratio. In the α1β2ε receptor, the GABA response ratio was 0.55 ± 0.14 (*n* = 5), indicating that 45% of receptors were blocked by picrotoxin. In contrast with reported findings of other GABA_A_ TM2-6’ mutants, the GABA response ratio in the α1β2ε(S296F) mutant was 0.12 ± 0.05 (*n* = 7), indicating increased sensitivity (88% blocked) to picrotoxin. The data are summarized in Figure 4D.

To confirm the incorporation of the ε(S296F) subunit in surface receptors, we compared sensitivity to Zn^2+^ in α1β2 and α1β2ε(S296F) receptors. In α1β2ε(S296F), co-application of 3 µM Zn^2+^ with 5 µM GABA (EC_58±11_) reduced the peak and residual currents at the end of a 30 s drug application to 77 ± 10% and 49 ± 8% (*n* = 5) of control, respectively. The observed inhibitory effect of Zn^2+^ on the peak response is significantly different (*p* < 0.01) in α1β2 and α1β2ε(S296F) receptors. The effect of Zn^2+^ on residual current is indistinguishable in α1β2 and α1β2ε(S296F) receptors.

As a positive control, we verified the effect of the γ2(T271F) (TM2-6’) mutation on inhibition by picrotoxin. Pre-application followed by co-application of 100 µM picrotoxin with 1 mM GABA reduced the GABA response ratio to 0.15 ± 0.09 (*n* = 7) in the α1β2γ2L receptor. In the α1β2γ2(T271F) receptor, the GABA response ratio was 0.85 ± 0.06 (*n* = 7), indicating reduced sensitivity to picrotoxin (Figure 4D).

In sum, the α1β2ε(L299S) receptor exhibits low P_A,constitutive_ while the α1β2ε(S296F) receptor retains high sensitivity to picrotoxin. Both findings are remarkably different from how mutations in homologous positions in other subunits modify GABA_A_ receptor function [34,35,36,37,38].

### 3.5. Accessibility of TM2 Residues in the ε Subunit

**Figure 5:**
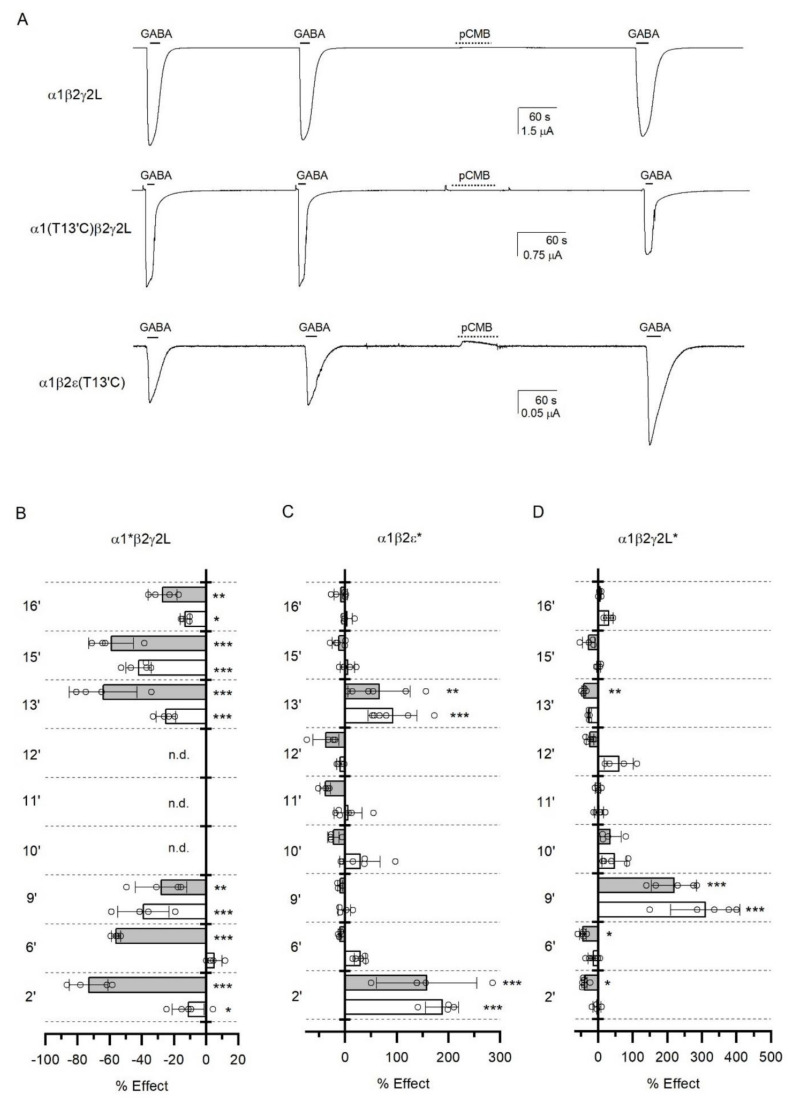
**Accessibility of residues in TM2 in α1, ε, and γ2L subunits.** (**A**) Sample traces showing the lack of effect of pCMB on response to GABA in the α1β2γ2L receptor (top trace), the inhibitory effect of pCMB on response to GABA in the α1(T13’C)β2γ2L receptor (middle trace), and potentiating effect of pCMB on response to GABA in the α1β2ε(T13’C) receptor (bottom trace). In each trace, the concentration of pCMB was 100 µM and the concentration of GABA was 1 mM. (**B**) Summary of effects of pCMB on α1*β2γ2L receptor (α1β2γ2L containing the TM2 mutations in the α1 subunit) function. (**C**) Summary of effects of pCMB on α1β2ε* receptor function. (**D**) Summary of effects of pCMB on α1β2γ2L* receptor function. In (**B**–**D**), the graphs show modulation of responses to saturating GABA upon exposure to pCMB alone (white) or pCMB + saturating GABA (grey). n.d., not done. Statistical tests were conducted using pairwise comparison of means with Dunnett’s correction. *, *p* < 0.05; **, *p* < 0.01; ***, *p* < 0.001.

Given the surprising effects of mutations of the 6’ and 9’ residues, in the next set of experiments we employed the scanning cysteine accessibility method to identify residues in TM2 of the ε subunit that line the ion channel. Residues in TM2 were individually mutated to cysteine, and the effect of exposure to the sulfhydryl-specific reagent pCMB, alone or coapplied with GABA, on peak response to saturating GABA was measured (Figure 5A). The overall goal was to determine the accessibility of ε-TM2 residues to pCMB and to identify any differences in the labeling of ε-TM2 and γ2L-TM2.

In initial control experiments, we measured the accessibility of selected α1-TM2 residues. The α1β2γ2L receptors containing cysteine substitutions in the α1 subunit (α1*β2γ2L) demonstrated significant changes in current responses to GABA upon labeling with pCMB at α1(I16’C) (residue number in mature peptide: 270), α1(S15’C), α1(T13’C), and α1(L9’C). Labeling was similar in the absence and presence of GABA, indicating that these residues are accessible to pCMB in resting and active/desensitized receptors. With the exception of modification of the current response in α1(S15’C), these data are in agreement with those previously reported [19]. We also tested the effect of labeling at α1(T6’C) and α1(V2’C). In agreement with a previous report [19], the α1(T6’C)- and α1(V2’C)-containing receptors were labeled with pCMB in the presence of GABA. Exposure to pCMB in the absence of GABA did not modify the current response in the α1(T6’C)β2γ2L receptor, and had a small, although statistically significant, effect in α1(V2’C)β2γ2L. In control experiments on the wild-type α1β2γ2L receptor, exposure to pCMB alone or pCMB in the presence of saturating GABA had no effect on response to subsequent application of GABA, indicating lack of modification of native cysteines. The data are summarized in Figure 5B.

Exposure to pCMB in the absence or presence of GABA modified current responses in the α1β2ε(T13’C) (mutated residue number in ε: 303) and α1β2ε(S2’C) receptors. No statistically significant modification of current response was observed in α1β2ε wild-type, α1β2ε(T16’C), α1β2ε(G15’C), α1β2ε(A12’C), α1β2ε(M11’C), or α1β2ε(T10’C). Notably, exposure to pCMB + GABA did not lead to modification of current responses in α1β2ε(L9’C) or α1β2ε(S6’C). One explanation for this is that the secondary structure of ε-TM2 differs from that of α1-TM2, rendering the TM2-9’ and TM2-6’ residues in the ε subunit inaccessible to pCMB. This would be consistent with our findings that the ε(L299S) (TM2-9’) and ε(S296F) (TM2-6’) mutations are ineffective at increasing sensitivity to GABA and decreasing sensitivity to picrotoxin, respectively, in the α1β2ε* receptor. An alternative explanation is that covalent labeling of cysteines at these positions in ε-TM2 does not result in a functional effect. The data are summarized in Figure 5C.

In the αβγ and αβδ ternary GABA_A_ receptors, the transmitter binding sites are formed at the interfaces between the two β/α subunit pairs. The fifth subunit, i.e., a γ or a δ, does not directly contribute to transmitter-induced activation, and at least in the related nicotinic receptor makes a smaller energetic contribution than the transmitter-binding subunits towards channel activation [39]. Due to their sequence homology, the ε subunit may substitute for the γ2 subunit in the ternary GABA_A_ receptor. We reasoned that if reduced involvement in channel activation underlies the lack of functional effect upon cysteine modification in ε-TM2, then an analogous pattern may be observed in the α1β2γ2L receptor in which modifications are made in γ2L-TM2.

We tested the effects of labeling with pCMB in α1β2γ2L(T16’C) (mutated residue number in γ2L: 281), α1β2γ2L(S15’C), α1β2γ2L(T13’C), α1β2γ2L(T12’C), α1β2γ2L(M11’C), α1β2γ2L(T10’C), α1β2γ2L(L9’C), α1β2γ2L(T6’C), and α1β2γ2L(L2’C) receptors. These data are summarized in Figure 5D. Significant effects were observed upon pCMB-modification in the α1β2γ2L(L9’C) receptor in the absence of GABA, and in the α1β2γ2L(T13’C), α1β2γ2L(L9’C), α1β2γ2L(T6’C), and α1β2γ2L(L2’C) receptors in the presence of GABA. Thus, the pattern of modification in γ2L-TM2 is more similar to that in α1-TM2 in the α1*β2γ2L receptor but differs from the pattern of modification in ε-TM2 in α1β2ε*. Most notably, pCMB-labeling of the TM2-9’ and TM2-6’ residues in the γ2L and α1 subunits, but not in the ε subunit results in functional effect.

## 4. Discussion

In this study, we have investigated the functional properties of the rat α1β2ε GABA_A_ receptor. We show that the receptor is inefficaciously activated by orthosteric and allosteric agonists, including the transmitter GABA, anesthetics propofol and pentobarbital, and the neurosteroid 3α5αP. Despite the remarkably high constitutive activity (P_A,constitutive_ = 0.18), the peak P_A_ in the presence of saturating GABA is only 0.71. Quantitative analysis of the current responses indicates that the binding of GABA provides −1.09 kcal/mol free energy change to stabilize the active state in the α1β2ε receptor, compared to ~−6 kcal/mol in the α1β2γ2L receptor. The anesthetics propofol and pentobarbital, and the neurosteroid 3α5αP are likewise low-efficacy agonists. The free energy changes in the presence of propofol and pentobarbital were −0.92 kcal/mol and −1.88 kcal/mol, respectively, which is 3–6-fold less than the ∆G in the α1β2γ2L receptor. The α1β2ε receptor was virtually unresponsive to 3α5αP (∆G = −0.06 kcal/mol).

Weak sensitivity of the ε-containing receptor to GABAergic neurosteroids may have physiological significance. During pregnancy, the levels of hormone-derived neurosteroids including 3α5αP increase by up to 100-fold [40], yet this is not accompanied by changes in respiratory function or behavior in the sedation spectrum. Conversely, it has been shown that pregnant rats exhibit a ~4-fold increase in the expression of the ε subunit in medullary neurons of the ventral respiratory column [41]. Thus, the increased expression of the ε subunit may be an adaptive response to increased neurosteroid concentrations in pregnancy to preserve normal physiological function.

A high level of constitutive activity has been observed in heterologously expressed αβε receptors [7,9,10,11,13]. In contrast, studies of ε subunit-expressing neurons in *locus coeruleus*, and hypothalamic and cardiac vagal neurons that express the ε subunit have reported no or minimal picrotoxin-sensitive constitutive activity [12,42,43]. The reason for this discrepancy is unclear.

Reduced sensitivity to allosteric agonists has been reported previously. Our data indicating a mere doubling of the response to low GABA in the presence of saturating 3α5αP is in excellent agreement with previous work on recombinant and native ε-containing receptors with neurosteroids [2,13,42]. Lower sensitivity to propofol and pentobarbital has been reported previously for recombinant ε-containing receptors [2,13]. Although direct comparison of the results is not trivial due to differences in the concentrations of GABA and/or the allosteric agonist, our data are in qualitative agreement with the previous reports. We note, however, that we did not observe significant differences in receptor properties upon overexpression of the ε subunit [13].

Transfection of rat cardiac parasympathetic neurons with the human ε subunit abolishes prolongation of sIPSCs in the presence of pentobarbital [12]. Likewise, the increased expression of the ε subunit in the medullary ventral respiratory column during torpor in hibernating 13-lined squirrel has been linked to resistance to pentobarbital-elicited depression of spontaneous neuronal activity [44,45]. Thus, the pharmacological properties of recombinant and native ε subunit-containing receptors are in agreement.

Previous studies of recombinant αβε receptors have employed a variety of subunit combinations including α1β1ε, α1β3ε, α2β1ε, α2β3ε, and α3β1ε [2,3,7,8,9,10,11,13]. To the best of our knowledge, this is the first study of the rat α1β2ε receptor. The reported properties of different subtypes of ε-containing receptors are generally consistent. The partners of the ε subunit in native receptors are not fully clear, although α3 and θ, or α3 and β1/3 have been proposed [42,46,47]. In HEK 293 cells, the ε subunit can co-assemble with α1β3γ2 subunits to form a functionally distinct αβγε receptor [48].

Studies of the effects of mutations of residues in TM2 provided some surprising results. The TM2-L9’S and TM2-S6’F mutations had atypical effects on receptor properties. The substitution of the conserved leucine at TM2-9’ in any subunit of the receptor normally leads to increased constitutive activity and sensitivity to agonists [34,35,36]. Introduction of the TM2-L9’S mutation to the α1 subunit α1β2ε receptor increased constitutive activity and sensitivity to GABA while in the ε subunit the mutation significantly reduced constitutive activity. Phenylalanine substitution at the TM2-6’ location decreases sensitivity to picrotoxin when introduced in α1, β2, or γ2 subunits [37,38]. However, when introduced in the ε subunit, the mutation significantly increased inhibition by picrotoxin. We note that receptors containing wild-type or mutated ε subunits showed reduced sensitivity to inhibition by Zn^2+^ compared to α1β2 receptors, an effect thought to be mediated by residues at the external end of TM2.

These findings motivated us to test the accessibility of TM2 residues to the cysteine-modifying reagent pCMB. The major finding of these experiments is that accessibility, as determined by changes in function, of ε-TM2 residues differs from that in α1-TM2 or γ2L-TM2. Most relevantly, changes in function upon exposure to pCMB were observed in receptors containing the TM2-L9’C mutation in α1 and γ2L subunits but not in the ε subunit. At this time, we are unable to distinguish between the two most parsimonious possibilities: that the ε subunit differs in its secondary structure in TM2, or that the labeling of TM2-L9’C in the ε subunit lacks functional effect. Based on a previous report of the γ2S subunit serving as an external accessory subunit to modulate receptor properties in addition to acting as an integral subunit of the GABA_A_ receptor [49], we note the possibility of the ε subunit acting solely as an external modulatory subunit to the α1β2 receptor in oocytes injected with cRNA for α1β2ε.

In sum, we have shown here that the constitutively active rat α1β2ε GABA_A_ receptor is inefficaciously activated by a number of orthosteric and allosteric agonists. Overexpression of the ε subunit was largely without effect on receptor properties. Results from mutagenesis and SCAM studies indicate significant differences in the properties of the TM2 domain in α1, γ2L, and ε subunits.

## Abbreviations

3α5αP: 5α-pregnan-3α-ol-20-one (allopregnanolone); GABA, γ-aminobutyric acid; P_A_, probability of being in the active state; P_A,constitutive_, constitutive probability of being in the active state; pCMB, 4-(hydroxymercuri)benzoic acid sodium salt; TM, transmembrane.
